# Influence of ischemia before vein grafting on early hyperplasia of the graft and the dynamic changes of the intima after grafting

**DOI:** 10.1186/1749-8090-7-90

**Published:** 2012-09-24

**Authors:** RongJiang Zou, MingJuan Sun, ZhiQian Lu, QingKui Guo

**Affiliations:** 1 Department of Cardiothoracic Surgery, Shanghai No6 People's Hospital, Shanghai JiaoTong University, 600 YiShan Road, Shanghai, 200233, China; 2 Department of Biochemistry and molecular biology, Second Military Medical University, Shanghai, Peoples Republic of China

**Keywords:** Vein graft, Ischemia, Intimal hyperplasia, Endothelial cell

## Abstract

**Background:**

To investigate both the influence of ischemia before grafting on early hyperplasia of the vein grafts, and the dynamic changes of the intima after grafting in a rabbit model of vein graft disease.

**Methods:**

We performed paired vein graft experiments under different ischemic conditions (15 vs. 60 min; 15 vs. 90 min) in the neck of the rabbits and compared the differences between the grafts. Clopidogrel, an anti-platelet agent, was administered before and after surgery. Twenty-eight days after the grafting procedure, the veins were evaluated microscopically. The dynamic changes of the intima after grafting were evaluated by scanning electron microscopy over time.

**Results:**

The vein grafts subjected to 60- or 90-min ischemia exhibited no differences compared to those subjected to 15-min ischemia in terms of the mean thickness of the intimal, medial, and adventitial layers of the graft. Similarly, there was no difference in the Ki-67 labeling index (proliferation marker) between the vein grafts. Vein grafts with 15-min ischemia lost endothelial cells (ECs) but healed by 3 days post graft, whereas vein grafts with 90-min ischemia suffered serious EC loss, which was restored with new ECs during days 2 to 14 post graft.

**Conclusions:**

Ninety-minute ischemia before vein grafting can cause serious EC loss, but does not increase early intimal hyperplasia when clopidogrel is administered. Protecting the vein from ischemia and reperfusion injury preserves ECs.

## Background

The autologous saphenous vein is the most common conduit for coronary artery bypass grafting (CABG), despite of increased use of arterial grafts in cardiac surgery. After grafting, this vein is subjected to immediate increases in flow, resulting in longitudinal wall shear stress, circumferential deformation, and pulsatile stress. This can cause intimal hyperplasia and progressive thickening of the vein graft wall to occur. Approximately 60% of vein grafts remain patent long term, of which only 50% are free of significant stenosis [1-3].

Endothelial cell (EC) injury plays a significant role in acute thrombosis after vein grafting [4]. Heparinized autologous blood and other solutions are typically used before grafting to protect the endothelium and its functions [5-7]. Another method for reducing acute thrombosis after grafting is the use of anti-platelet drugs to reduce the risk of thrombosis [8]. Such drugs have been demonstrated to improve graft patency and have been used routinely post operation [9]. When the risk of acute of thrombosis is reduced by such means, influence of ischemia before vein grafting on early hyperplasia of the graft can be studied to find out the ischemic time not increasing the hyperplasia under different preservation. In this study, we designed a series of paired trials to evaluate the effects of different ischemic times on hyperplasia associated with vein grafts when clopidogrel, an anti-platelet agent, was used.

## Methods

### Animals and grouping

New Zealand white rabbits weighed between 2.5 and 3.0 kg were raised and provided by the Laboratory Animal Center of Shanghai No. 6 People's Hospital. The protocol for animal experiments was approved by the Committee of Ethics on Animal Experiments at the Shanghai Jiao Tong University School of Medicine, based on the Guidelines for Animal Experiments. The rabbits were randomly divided into two groups: (1) 15- vs. 60-min ischemia and (2) 15- vs. 90-min ischemia.

### Vein graft surgery

Anesthesia was induced in the rabbits with intravenous ethaminal sodium (15 to 30 mg/kg, depending on the response of the rabbit to the drug), allowing spontaneous ventilation throughout the procedure. In addition, heparin sodium (250 U/kg) and penicillin (400 kU) were administered intravenously before creating a skin incision. In each animal, a longitudinal incision was made in the neck over the region of the internal jugular vein. The internal jugular vein and the common carotid artery were dissected using the “no touch” technique, and the side branches were ligated with 5–0 silk sutures.

**Figure 1:**
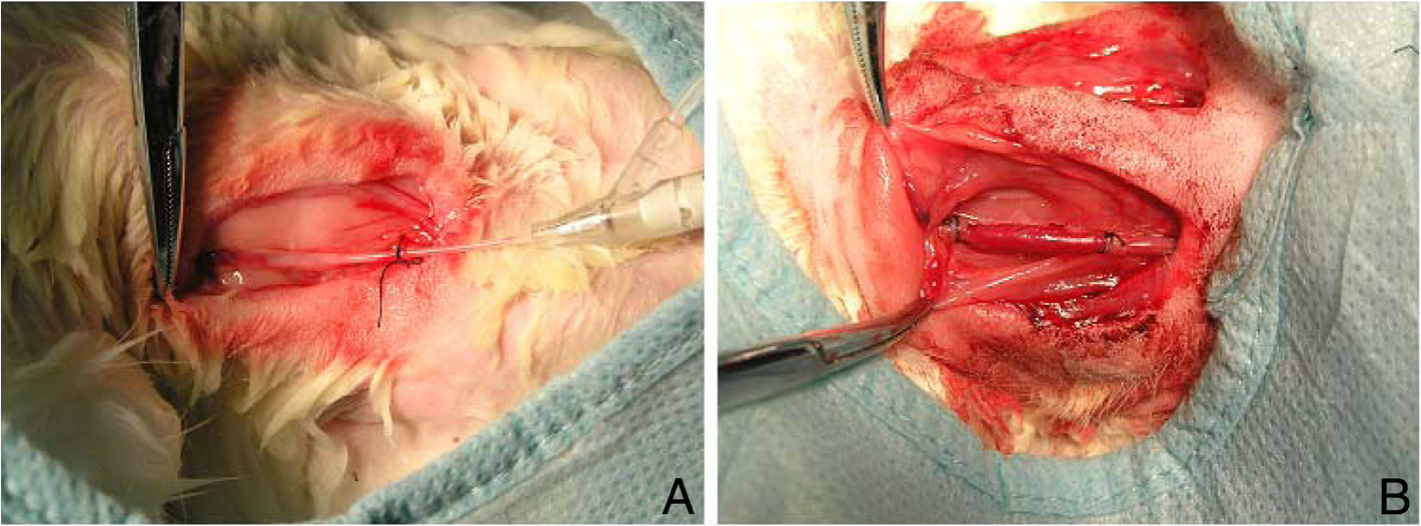
**Surgery.** The vein was punctured with a intravenous remained trocar of 22G and was ligated on it (left image). Subsequently, the vein and trocar were removed. The infusing process was very simple and unproblematic, the vein did not spasm, and the procedure was carried out quickly (right image).

To avoid the operating difficulty caused by the spasm of vein, a intravenous remained trocar of 22 G was inserted into the distal internal jugular vein and secured in place with a ligature (Figure 1). A 2-cm portion of the internal jugular vein including the trocar was removed and rinsed with saline, then placed in saline containing heparin sodium (62.5 U/ml, 20°C) for 45 (group 1) or 75 min (group 2). A 1-cm segment of the common carotid artery between two vascular clamps was also removed. Polyvinyl chloride cuffs with 1-mm inner diameter were fixed to each end of the artery, around which the artery was everted and ligated. Subsequently, the vein with little protection in saline was sleeved over the cuffs and ligated. When the entire ischemic time was 60 (group 1) or 90 min (group 2), the vascular clamps were removed, pulsations and turbulent blood flow within the vein indicated successful grafting. On the contralateral side, a similar procedure was performed except the vein was sleeved immediately after being removed, and the entire ischemic time was 15 min. All animals received bilateral grafts to establish a paired comparison in the two groups consisting of 6 rabbits each (15- vs. 60-min ischemia; 15- vs. 90-min ischemia). Postoperatively, the rabbits were housed individually at 20°C and fed a normal diet with free access to water. Clopidogrel was administered to each rabbit the day before the surgery (6 mg/kg) and daily after surgery for 4 weeks (3 mg/kg).

After 4 weeks, the grafts, each including a 0.5-cm segment of the proximal and distal common carotid artery, were harvested from the rabbits under anesthesia. The grafts were rinsed in saline and the vessel lumen was infused with 10% formalin at a pressure of 100 mmHg, simulating the systolic pressure for 12 h. Each of the grafts was divided into three equal parts. Each part was dehydrated, cleared, and paraffin-embedded. Approximately 5-μm transverse sections were taken from three points in each graft, mounted onto glass slides, and stained with hematoxylin/eosin (H/E) and Ponceau Red/Victoria Blue (P/VB).

To evaluate the influence of the infusion pressure on the intima by scanning electron microscopy (SEM), two sides of the ungrafted internal jugular veins were removed from 2 rabbits. After being rinsed in saline, the veins of one side were preserved in 2.5% glutaraldehyde (SIGMA, St. Louis, MO) for 12 h, and the veins of the other side were infused intraluminally with 2.5% glutaraldehyde at a pressure of 100 mmHg for 12 h. All veins were bisected and secured on a cork mounting board with 8–0 polypropylene sutures.

To observe dynamic changes in the intima by SEM, an additional 15- vs. 90-min ischemia group was also studied. Grafts were harvested at 1 h, and 1, 2, 3, 7, 14, and 28 days after grafting (2 rabbits at each time point). These grafts were rinsed in saline and infused intraluminally with 2.5% glutaraldehyde at a pressure of 100 mmHg for 12 h. Subsequently, the grafts were bisected and secured on a cork mounting board with 8–0 polypropylene sutures.

### Morphometric analysis

The vessel wall dimensions were captured under an LW200T light microscope (Shanghai Cewei Photoelectric Technology Co. Ltd., Shanghai, China) with a color video camera head (DH-HV3103UCUSB; Daheng Group, Inc., Beijing, China) and measured with YRMV image-analysis software (Shanghai Yinrui Information Technology Co. Ltd., Shanghai, China). The mean intimal, medial, and adventitial thickness measurements were derived by measuring the areas and perimeters of the borders between the intimal and medial sections of each graft.

### Immunocytochemistry and cell proliferation analysis

Cell proliferation was detected with immunohistochemistry using Ki-67 via a 3-step staining procedure. Briefly, 5-μm-thick sections were cut from all paraffin-embedded tissue samples, placed onto slides, dewaxed, and hydrated in graded alcohols. Microwave pretreatment with BD Retrievagen A (pH 6.5) (BD Biosciences, San Diego, CA) for 10 min was performed, and slides were left to cool for 30 min before being rinsed in 0.05 mol/L Tris-buffered saline (TBS). Sections were then incubated with rabbit serum for 30 min, followed by a 1-h incubation with mouse monoclonal antibody against Ki-67 (B56; BD Biosciences). Subsequently, sections were treated with a secondary biotinylated goat anti-mouse antibody (DAKO, Glostrup, Denmark) for 10 min. TBS was used as a washing buffer between the antibody incubation steps. The samples were then incubated with streptavidin-HRP (DAKO, Glostrup, Demmark) for 10 min and 0.05% 3,3-diaminobenzidine (DAB) for 10 min. A light counter stain with hematoxylin (30 sec) was applied to permit visualization of morphology.

In each tissue section, the total number of cells and the number of Ki-67-positive cells were counted in the intima, media, and adventitia in 6 microscopic fields of view with a 40× objective. The labeling index was calculated as the percentage of positive nuclei.

### Scanning electron microscopy

Samples were fixed in 2.5% glutaraldehyde, rinsed in 0.1 M phosphate buffer, fixed in 1% osmic acid (4°C for 2 h), and dehydrated through a graded series of ethanol (50% to 100%) and amyl acetate. The samples were critical point-dried (HCP-2; Hitachi, Tokyo, Japan), sputter coated with a thin layer of gold (IB-3; Eiko Engineering, Ibataki, Japan), and analyzed with SEM (S-520; Hitachi).

### Statistical analysis

All data were analyzed using SPSS 12.0 for Windows, and are expressed as the mean ± standard deviation (SD). A comparison between veins with different ischemic times in each group was performed using the paired Student’s *t*-test. *P*-values less than 0.05 were considered statistically significant.

## Results

### Graft patency

All grafts remained patent after operation, and no cases of grossly visible thrombus or mural thrombus occurred. The grafts were adherent to surrounding tissues, and some regions were seemingly well incorporated.

### Morphometric analysis

**Figure 2:**
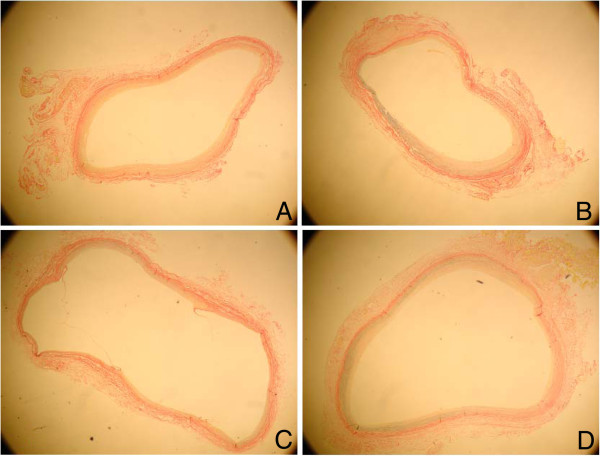
**Histological appearance of the grafts (stained with P/VB; magnification ×3) 4 weeks after the grafting procedure.A**: vein grafts with 15-min ischemia (group 1); **B**: vein grafts with 60-min ischemia (group 1); **C**: vein grafts with 15-min ischemia (group 2); **D**: vein grafts with 90-min ischemia (group 2).

Using the mean thickness of the 3 layers to compare graft hyperplasia, we found no differences between vein grafts with 15- and 60-min ischemia or 15- and 90-min ischemia at 4 weeks after the procedure (Table 1, Figure 2).

**Table 1:** Comparison of vein grafts under different ischemic conditions

**Thickness of graft layers (μm)**	**Group 1**		**Group 2**	
	**15-min ischemia**	**60-min ischemia**	**15-min ischemia**	**90-min ischemia**
Intima	50 ± 13	45 ± 6	54 ± 10	62 ± 21
Media	51 ± 8	40 ± 13	52 ± 3	52 ± 10
Adventitia	219 ± 39	188 ± 52	183 ± 23	253 ± 61

Values are mean ± standard deviation.

### Immunocytochemistry and cell proliferation analysis

**Figure 3:**
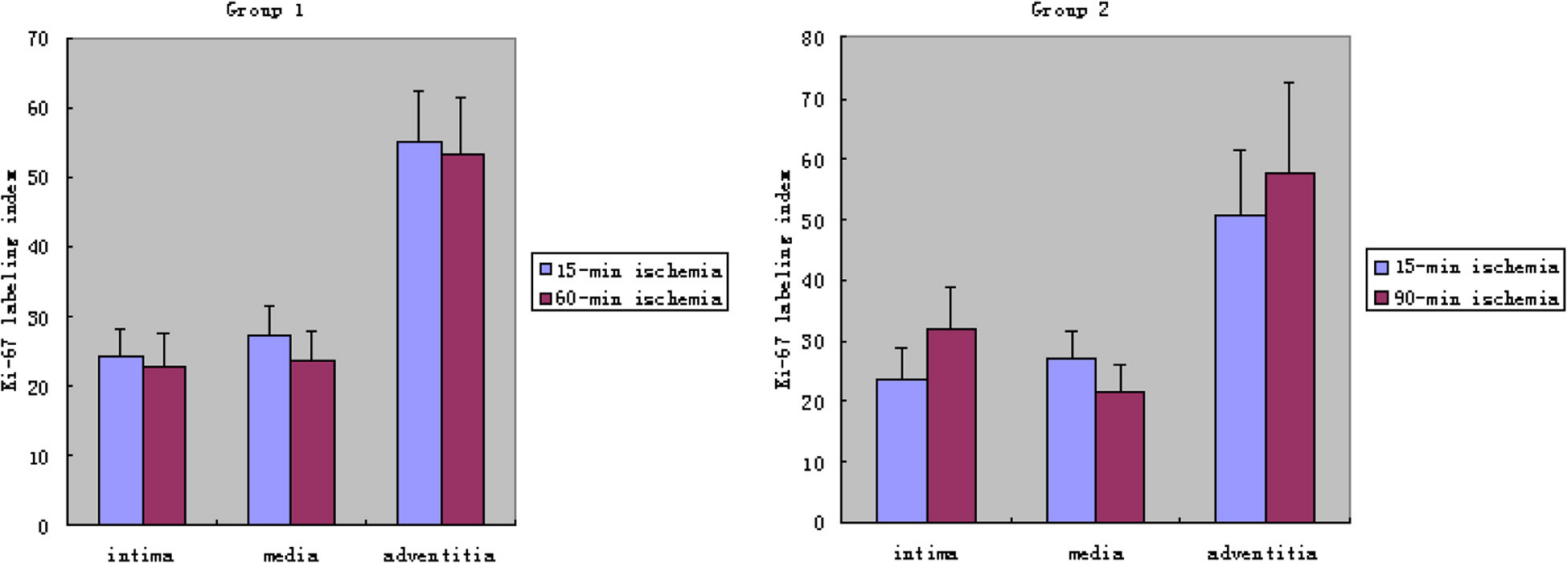
The Ki-67 labeling index of the vein grafts.

Many Ki-67-positive cells were found in the layers of the vein grafts, especially in the adventitia. There were no significant differences between the vein grafts that underwent different ischemic times in terms of the labeling index (Figure 3), as in the mean thickness results.

### Scanning electron microscopy observation of intima

**Figure 4:**
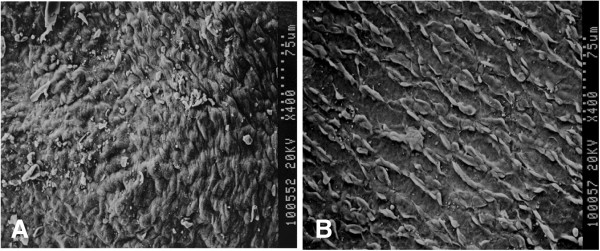
**Scanning electron micrographs of the intima surface of an ungrafted vein (original magnification ×400).A**: uninfused vein; **B**: infused vein.

SEM revealed that the intima of the ungrafted vein maintained its integrity before infusion but was notably torn after infusion (Figure 4), likely due to the infusion pressure.

**Figure 5:**
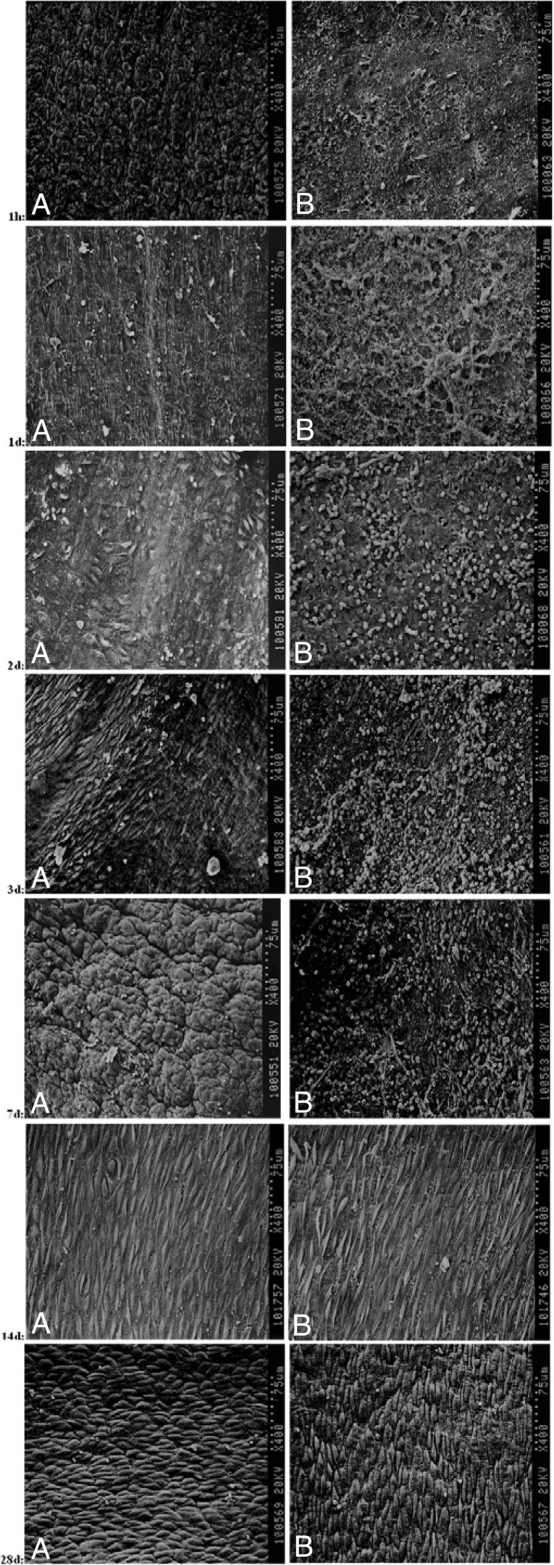
**Scanning electron micrographs of the intima surface of grafted veins (original magnification ×400) after 1 h and 1, 2, 3, 7, 14, or 28 days.A**: vein grafts with 15-min ischemia; **B**: vein grafts with 90-min ischemia.

One hour after grafting, changes in the vein with 15-min ischemia included a widely torn intima with no loss of ECs. In the vein with 90-min ischemia, however, all ECs were lost, though the sub endothelial collagen fibers were not exposed. After 24 h, the intima of vein with 15-min ischemia showed little change, while in the vein with 90-min ischemia collagen fibers were thoroughly exposed. After 2 days, the vein with 15-min ischemia lost a portion of its ECs, which were disorderly arranged. In addition, the vein with 90-min ischemia began to show signs of restoration, with the collagen fibers covered by new ECs. The surface also showed infiltration of white blood cells (WBCs) and red blood cells (RBCs). Three days after grafting, the intima of vein with 15-min ischemia regained integrity, the ECs arranged orderly, whereas the intima of vein with 90-min ischemia had adherent surface fibrin and micro thrombi. After 7 days, the intima integrity of the vein with 15-min ischemia persisted and included folds, likely caused by hyperplasia. Similarly, the intima of the vein with 90-min ischemia also had folds, in addition to adherent fibrin and micro thrombi. After 14 days, both intimae showed integrity, including orderly arranged ECs and no fibrin adherence, WBCs, or RBCs. Twenty-eight days after grafting, both intimae with crowded ECs presented a “cobblestone” appearance (Figure 5).

## Discussion

Ischemic injury before grafting and reperfusion injury after grafting may injure ECs, and subsequently activate platelets and trigger the coagulation cascade [10]. Various solutions and heparinized autologous blood have been used previously for vein preservation to reduce ischemia and reperfusion (IR) injury. For instance, Cavallari et al. used autologous whole blood (AWB), 0.9% normal saline solution (NS), and University of Wisconsin solution (UWs) individually to treat autogenous vein grafts for 45 min at 4°C before grafting, and found the veins stored in UWs had a similar intimal thickness with that of control samples, whereas the veins stored in AWB or NS had a thicker intima layer after grafting [11]. However, Solberg et al. found that veins stored in cell culture media at 4°C show a marked increase in EC dysjunction and an 18% loss of EC number, compared to only a 4% loss at 20°C, indicating that hypothermy may injure ECs [12]. In typical situations, the veins are placed in saline containing heparin sodium at 20°C after being removed. Therefore, we designed this study to investigate the influence of pre-grafting ischemia on early hyperplasia after grafting under these conditions, and found the ischemic tolerance of the vein. After the step, we planed to investigate how long the veins can be preserved in the solutions (such as UWs) or how long the AWB should be renewed. The cuff technique was used in this study for the vein graft model because it can limit the ischemic time caused by the procedure to 15 min, avoiding IR injury. In one side, the veins were grafted immediately after removal to offer controls, whereas, in the other side, the veins were stored before being used. We also included clopidogrel treatment to reduce the risk of thrombus or mural thrombus and to make the vein graft model more similar to the clinical situation.

Prospective controlled trials have demonstrated a graft patency benefit when aspirin was started 1, 7, or 24 h postoperatively, a benefit which was lost when started after 48 h [9]. Aspirin has been shown to significantly reduce postoperative mortality, myocardial infarction, stroke, renal failure, and bowel infarction when administered within 48 h of CABG [8]. However, in patients with atherosclerotic vascular disease, clopidogrel is more effective than aspirin in reducing the combined risk of stroke, myocardial infarction, and vascular death [13].

Besides having anti-thrombotic properties, inhibitors of platelet aggregation attenuate platelet function, prevent platelet activation and release of peptide growth factors and nonpeptide substances, and thereby inhibit intimal hyperplasia [14]. Atherosclerosis is described as an inflammatory disease [15]. Clopidogrel inhibits the expression of platelet activation markers and the interaction of platelets and leukocytes [16], and is powerful in decreasing the levels of inflammatory factors [17].

In our present study, SEM revealed that 90-min ischemia caused serious injury of the intima, including the exposed subendothelial collagen fibers that may aggregate and activate platelets and the frequently adherent leukocytes, which may trigger vascular inflammation. Clopidogrel was used and avoided thrombus or mural thrombus successfully. Moreover, results show no differences between vein grafts with 15- and 60-min ischemia or 15- and 90-min ischemia in term of thickness and the hyperplasia intensity of the intima, media, or adventitia at 4 weeks after the grafting procedure. It seems ischemia up to 90 min does not increase early intimal hyperplasia of the vein graft when clopidogrel was used effectively. Approximately 90 min is a long enough time period for surgeons to complete 3 or 4 bypasses; therefore, when using clopidogrel, it is seemingly unnecessary to consider the influence of ischemia before grafting on early hyperplasia of the vein grafts.

The recovery of the intima was also observed post operation. EC coverage has been used in some studies [18,19], which is easily gained and facilitates statistical analysis. However, based on our study, it is apparently unreliable because fibrin and micro thrombi covered the surface of the intima when there was IR injury according to SEM analysis. Therefore, we only reported the characteristics of the intima in this study.

According to our observations, the intimae of vein grafts with 15-min ischemia only lost a portion of their ECs and reintegrated at 3 days post operation. However, the intimae of vein grafts with 90-min ischemia lost all their ECs and redintegrated at 14 days post operation. Furthermore, the anterior had less risk of thrombosis. Reducing ischemia and reperfusion injury is valuable. It has been suggested previously that UWs preserves the vein before grafting because of its excellent effect on protecting the endothelium and its functions [20].

However, vein grafts with 15-min ischemia, which had almost no IR injury, likely face a widely torn intima caused by systolic pressure 1 h after grafting. Furthermore, the vein grafts with 90-min ischemia likely face more serious loss of ECs until new ECs are present, which come predominantly from extrinsic cells such as bone marrow-derived cells [21], and form an intima layer with integrity. Thus, it seems antithrombotic therapy should be administered as early as possible post operation.

Gavaghan et al. found that administering aspirin within 1 h of CABG did not increase chest-tube blood loss, the red cell transfusion requirements, or the re-exploration rate [22]. However, Goldman et al. reported that aspirin use on the day before CABG has a similar effect on early graft patency within 6 h after surgery, and increased bleeding complications [23]. Considering that the number of platelets and the levels of blood coagulation factors vary and change with time after CABG, we recommend starting anti-platelet therapy when the postoperative chest tube drainage is ≤50 ml/h for 2 consecutive hours, as presented by both Halkos et al. and Chan et al. [24,25].

## Conclusions

In summary, our study indicates that, when clopidogrel is administered effectively, ischemia up to 90 min does not increase early intimal hyperplasia of the vein grafts, although a long-term study is necessary for information on later time points after operation. Furthermore, IR injury can cause serious EC loss, avoiding the injury preserves ECs.

## Competing interest

The authors declare that they have no competing interests.

## Authors' contributions

RJZ carried out the whole study and drafted the manuscript. MJS: carried out the morphometric analysis, immunocytochemistry and cell proliferation analysis, scanning electron microscopy analysis. ZQL: designed and supported the study, revised the manuscript. QKG: carried out the surgery. All authors read and approved the final manuscript.
